# Assessing invasiveness of subsolid lung adenocarcinomas with combined attenuation and geometric feature models

**DOI:** 10.1038/s41598-020-70316-3

**Published:** 2020-09-03

**Authors:** Constance de Margerie-Mellon, Ritu R. Gill, Pascal Salazar, Anastasia Oikonomou, Elsie T. Nguyen, Benedikt H. Heidinger, Mayra A. Medina, Paul A. VanderLaan, Alexander A. Bankier

**Affiliations:** 1 Department of Radiology, Beth Israel Deaconess Medical Center, Harvard Medical School, Boston, MA USA; 2 Vital Images, Minnetonka, MN USA; 3 Department of Medical Imaging, Sunnybrook Health Sciences Centre, University of Toronto, Toronto, Canada; 4 Department of Medical Imaging, Toronto General Hospital, University of Toronto, Toronto, Canada; 5 Department of Biomedical Imaging and Image-Guided Therapy, Vienna General Hospital, Medical University of Vienna, Vienna, Austria; 6 Department of Pathology, Beth Israel Deaconess Medical Center, Harvard Medical School, Boston, USA; 7 Department of Radiology, UMass Memorial Medical Center, University of Massachusetts Medical School, Worcester, MA USA

**Keywords:** Cancer imaging, Cancer, Predictive markers, Cancer imaging, Lung cancer

## Abstract

The aim of this study was to develop and test multiclass predictive models for assessing the invasiveness of individual lung adenocarcinomas presenting as subsolid nodules on computed tomography (CT). 227 lung adenocarcinomas were included: 31 atypical adenomatous hyperplasia and adenocarcinomas in situ (class H1), 64 minimally invasive adenocarcinomas (class H2) and 132 invasive adenocarcinomas (class H3). Nodules were segmented, and geometric and CT attenuation features including functional principal component analysis features (FPC1 and FPC2) were extracted. After a feature selection step, two predictive models were built with ordinal regression: Model 1 based on volume (log) (logarithm of the nodule volume) and FPC1, and Model 2 based on volume (log) and Q.875 (CT attenuation value at the 87.5% percentile). Using the 200-repeats Monte-Carlo cross-validation method, these models provided a multiclass classification of invasiveness with discriminative power AUCs of 0.83 to 0.87 and predicted the class probabilities with less than a 10% average error. The predictive modelling approach adopted in this paper provides a detailed insight on how the value of the main predictors contribute to the probability of nodule invasiveness and underlines the role of nodule CT attenuation features in the nodule invasiveness classification.

## Introduction

Persistent subsolid nodules detected on thoracic computed tomography (CT) examinations commonly represent cancers belonging to the lung adenocarcinoma spectrum^1−3^. Correctly identifying the degree of histological invasiveness of a cancer from this spectrum is important because it may directly impact management, such as the length of CT follow-up intervals and the anatomical extent of surgery, with resection of more lung parenchyma for lesions with a high degree of invasive morphological features, for example by lobectomy, versus resection of less lung parenchyma for lesions with a lesser degree of invasive morphological features, for example by wedge resection^4^. To overcome the variability inherent to assessing invasiveness with subjective and qualitative parameters, several models based on objective and quantitative parameters have recently been investigated^5−10^. In particular, parsimonious logistic regression models using both the nodule volume and features extracted from the CT histogram performed well in predicting the probability of invasiveness^11^.

By classifying nodules as either non-invasive or invasive adenocarcinomas, however, these models were limited by their binary output. Because this binary output hardly reflects the histological complexity of the adenocarcinoma spectrum, we aimed to expand this binary into a multiclass model classification that would better match the current histological adenocarcinoma categories^3^. Being a natural multiclass extension of the logistic regression used in binary classification, we assumed that ordinal regression models would provide incremental information about how CT input features would affect outcome categories of degrees of invasiveness. If successful, this approach would enable radiologists to assess the probability of invasiveness of individual cancers as defined by CT characteristics of lung nodules. The aim of our study, therefore, was to develop and test multiclass predictive models based on ordinal regressions for assessing the invasiveness of individual lung adenocarcinomas presenting as subsolid nodules on CT examinations.

## Results

### Descriptive analysis

#### Patient and CT protocol characteristics

A total of 227 nodules were used in this study. Overall, 31 nodules were of type H1 (atypical adenomatous hyperplasia AAH, N = 1, and adenocarcinoma in situ AIS, N = 30), 64 nodules were of type H2 (minimally invasive adenocarcinoma MIA) and 132 nodules were of type H3 (invasive adenocarcinoma). Table 1 summarizes the patient and CT protocol characteristics of the dataset. These characteristics did not differ significantly between the classes (Supplementary Table S1* online*).

**Table 1:** Patient and CT protocol characteristics, and geometric and attenuation features for the 3-class nodule classification.

	All (n = 227)	H1 (n = 31)	H2 (n = 64)	H3 (n = 132)
**Age (years)**	67 ± 10	66 ± 10	66 ± 10	67 ± 9
**Sex**				
Male	60 (26%)	5 (16%)	19 (30%)	36 (27%)
Female	167 (74%)	26 (84%)	45 (70%)	96 (73%)
**Smoking**				
No	63 (28%)	12 (39%)	15 (23%)	36 (27%)
Yes	164 (72%)	19 (61%)	49 (77%)	96 (73%)
**CT section thickness**				
1.0–1.5 mm	122 (54%)	13 (42%)	40 (62%)	69 (52%)
2.0–2.5 mm	16 (7%)	1 (3%)	2 (3%)	13 (10%)
3.0 mm	89 (39%)	17 (55%)	22 (34%)	50 (38%)
**Location**				
Right upper lobe	77 (34%)	15 (48%)	19 (30%)	43 (33%)
Right middle lobe	12 (5%)	2 (6%)	2 (3%)	8 (6%)
Right lower lobe	37 (16%)	1 (3%)	15 (23%)	21 (16%)
Left upper lobe	69 (30%)	10 (32%)	22 (34%)	37 (28%)
Left lower lobe	32 (14%)	3 (10%)	6 (9%)	23 (17%)
**CT geometric features**				
Average diam. (mm)	17.9 (12.9, 23.0)	13.4 (10.9, 16.5)	14.8 (11.9, 20.4)	20.1 (16.0, 25.1)
Max. diam. (mm)	21.0 (15.0, 28.4)	16.0 (12.1, 20.0)	17.8 (14.0, 23.8)	24.3 (18.5, 31.7)
Min. diam. (mm)	13.1 (10.5, 18.7)	11.2 (8.7, 13.0)	12.0 (9.2, 17.4)	15.2 (11.9, 19.7)
Max.min.diam. ratio	1.5 (1.3, 1.8)	1.5 (1.2, 1.7)	1.4 (1.3, 1.7)	1.6 (1.3, 1.9)
Consolidation ratio	0.43 (0.00, 0.60)	0.13 ± 0.18	0.31 ± 0.27	0.52 ± 0.31
Volume (mm3)	2,242 (1,099, 5,154)	1,362 (458, 2,178)	1,297 (830, 4,088)	3,436 (1,710, 6,349)
Volume (log)	3.4 ± 0.49	3.1 ± 0.49	3.2 ± 0.44	3.5 ± 0.46
**CT attenuation features**				
Mean (HU)	−500 ± 155	−649 ± 106	−569 ± 113	−432 ± 143
Standard deviation (HU)	200 ± 55	146 ± 40	173 ± 47	225 ± 45
Skewness	0.48 (0.15, 0.78)	0.61 (0.40, 0.92)	0.68 (0.38, 0.91)	0.32 (-0.04, 0.55)
Kurtosis	2.7 (2.1, 3.5)	3.5 (2.8, 4.4)	3.2 (2.6, 4.2)	2.4 (2.0, 2.9)
IQR (HU)	282 (200, 381)	173 (142, 210)	228 (172, 297)	354 (267, 437)
Q.50 (HU)	−552 (−655, −419)	−702 (−787, −612)	−610 (−682, −534)	−486 (−582, −356)
Q.75 (HU)	−374 (−515, −205)	−593 (−678, −438)	−464 (−561, −367)	−272 (−400, −108)
Q.875 (HU)	−261 ± 220	−542 (−619, −380)	−368 (−496, −257)	−136 (−282, −16)
FPC1	0.00 ± 0.37	−0.37 (−0.48, −0.17)	−0.19 (−0.40, 0.03)	0.16 (−0.02, 0.42)
FPC2	−0.00 ± 0.21	−0.07 ± 0.16	0.01 ± 0.17	0.01 ± 0.23

Normally distributed continuous variables are shown as mean ± standard deviation, non-normally distributed features as median (interquartile range). Categorical variables are shown as number (%). Results of the comparison of patient and CT protocol characteristics, and geometric and attenuation features for the 3 classes of nodules are presented in Supplementary Table S1.

H1: atypical adenomatous hyperplasia and adenocarcinoma in situ.

H2: minimally invasive adenocarcinoma.

H3: invasive adenocarcinoma.

Max.: maximum, Min.: minimum, Diam.: diameter.

Max.min.diam. ratio : maximum diameter/minimum diameter.

Consolidation ratio: solid component maximum diameter/nodule maximum diameter.

HU: Hounsfield unit.

Q.50: CT attenuation value at the 50th percentile, Q.75: CT attenuation value at the 75th percentile, Q.875: CT attenuation value at the 87.5th percentile.

#### CT geometric and CT attenuation features

Most CT geometric and CT attenuation features showed significant differences in mean or median values between classes. All numerical values for these features are shown in Table 1, and results of the statistical comparisons between the 3 classes are described in Supplementary Table S1 online.

**Figure 1:**
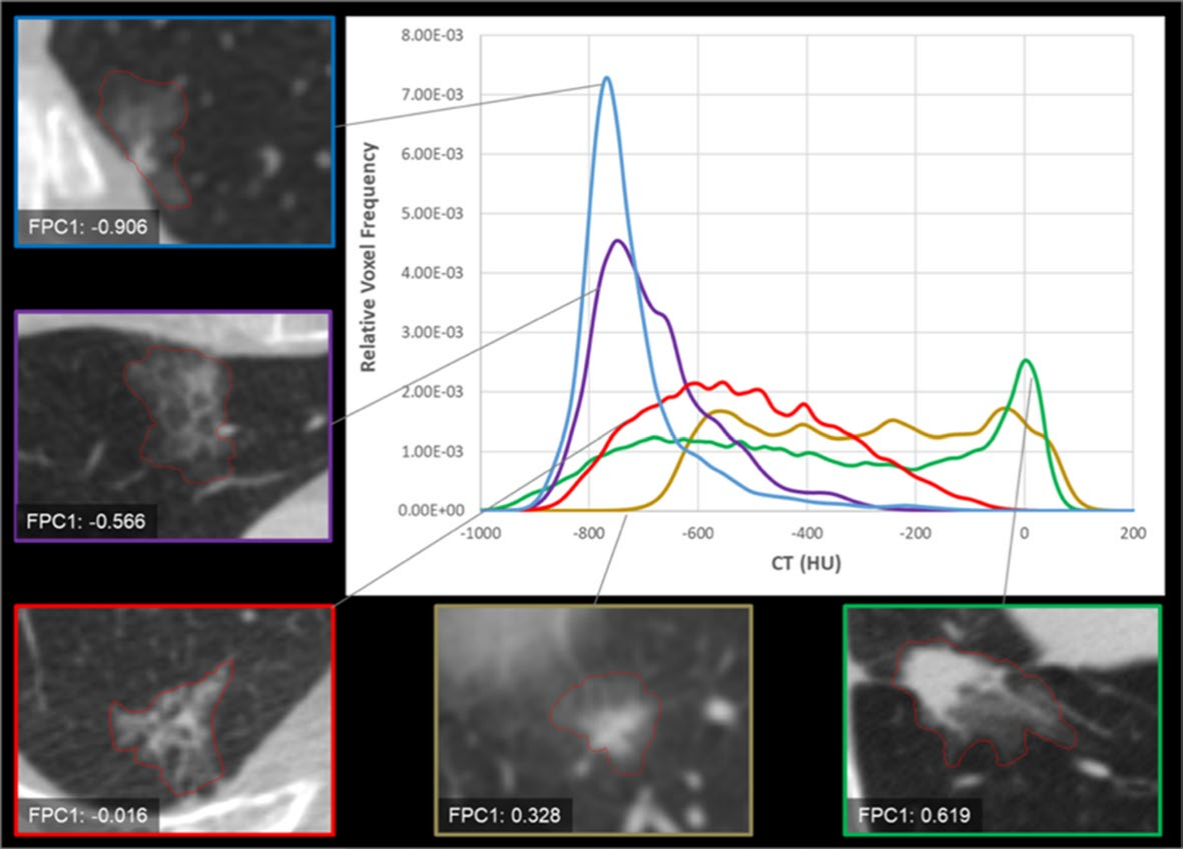
CT attenuation curves of five nodules. It shows the CT attenuation curves for a selection of five subsolid nodules with increasing FPC1 values from FPC1: -0.906 to FPC1: 0.619. When FPC1 values increase, the CT attenuation curves become more heterogeneous corresponding to an increasing number of voxels with higher CT attenuation within the nodule.

The two first components generated from the functional principal component analysis were called FPC1 and FPC2 and were found to account for 83% of the attenuation curve variation. These data-driven attenuation features were retained for further use as new continuous features summarizing the two main modes of variation of the CT attenuation curves in the dataset. The first component FPC1 accounted for 63% of the attenuation curve variation across the dataset. A sample of five subsolid nodules with increasing FPC1 values and their respective CT attenuation curves is shown in Fig. 1. The second component FPC2, accounting for 20% of the curve variation in the dataset was not found to be significantly different among the 3 nodule classes and was not retained for further analysis.

### Model building with ordinal regression

#### Feature selection

**Figure 2:**
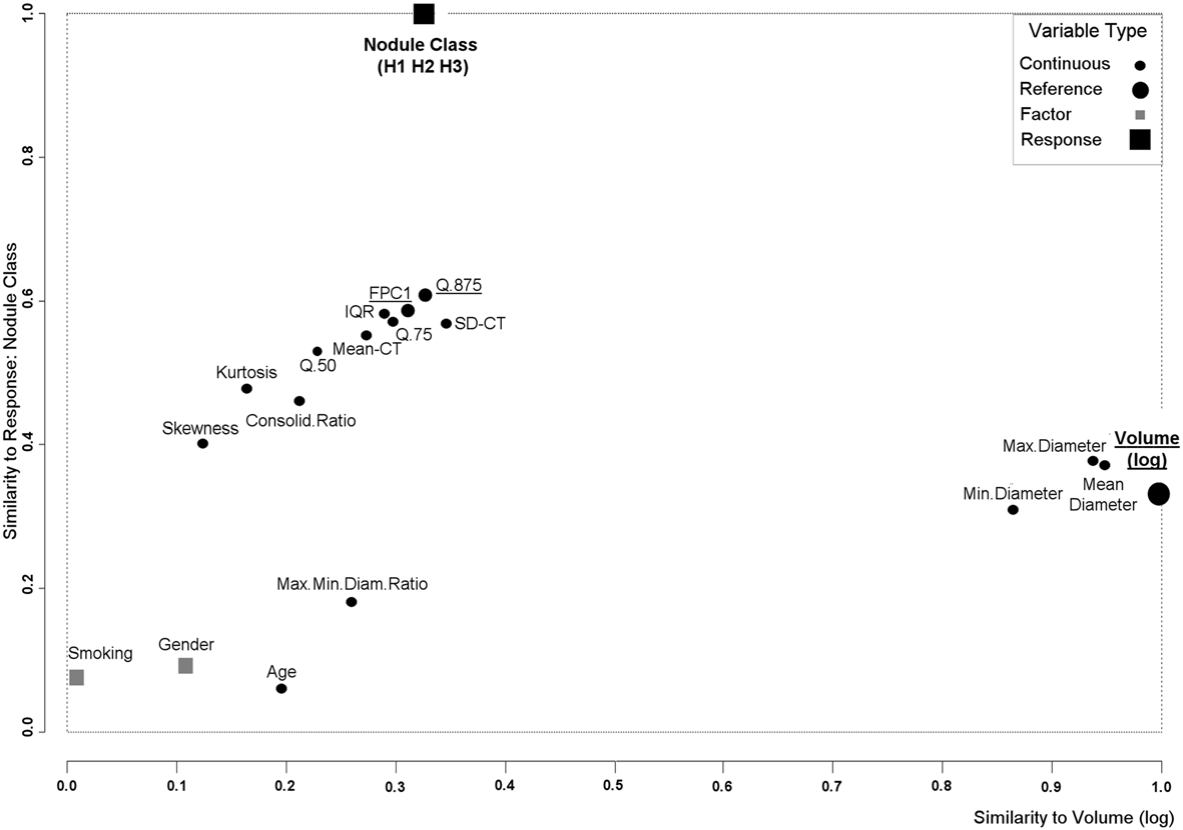
Confounder plot for feature selection. Candidate features are plotted using on the y-axis the similarity to the response (nodule class H1, H2 or H3) and on the x-axis, the similarity to the reference main predictor (nodule volume (log)). Best features are on the top-left side of the plot. Right-sided predictors are more correlated with the volume (log) and may be excluded to avoid collinearity among the predictive features.

Table 2 presents the ROC-AUC performance of individual features with AUCs significantly higher than 0.5 (*P* < 0.001) for the nodule classification H1 or H2 vs H3 in the full dataset. The nonparametric and FPC1 CT attenuation features had the highest AUCs (0.82–0.84), followed by the parametric attenuation features (0.77–0.82) and geometric features (0.61–0.75). Similarly, Table 3 shows the ROC-AUC performance of individual features with AUCs significantly higher than 0.5 (*P* < 0.001) for the nodule classification H1 vs H2 or H3 in the full dataset. Built on a distinct approach that relies on variable similarity, the confounder plot shown in Fig. 2 uses the three nodule classes (H1, H2, H3) as the response and the nodule volume (log) (logarithm of the nodule volume) as the reference feature. The nodule volume (log) was selected as the reference feature to include in our models for its overall characteristics: its AUCs in univariate test (AUC = 0.71 for H1 or H2 vs H3 classification and AUC = 0.70 for H1 vs H2 or H3 classification), its known linearity with the response and its low inter-user variability^11^. The CT attenuation features FPC1 and Q.875 (CT attenuation value at the 87.5% percentile) had the highest association with the response (hence the highest predictive values) and each of them was only modestly correlated with the reference feature volume (log). Therefore, they were the best candidate features for model building. The other CT attenuation features had a lower predictive value and were therefore excluded from further analysis. Diameter-type features were highly correlated with volume (log) and were also excluded from further analysis to avoid multi-feature collinearity that would reduce the performance of our models.

**Table 2:** Main features and ROC-AUC performances for H1 or H2 vs H3 nodule classification (full dataset).

Parameter	AUC (95% CI)	Sensitivity	Specificity	Best threshold
Q.875 (HU)	0.84 (0.78; 0.88)	71	84	> −253
FPC1	0.83 (0.78; 0.88)	67	90	> 0.073
IQR (HU)	0.83 (0.76; 0.87)	76	74	> 265
Q.75 (HU)	0.82 (0.77; 0.87)	74	79	> −393
SD (HU)	0.82 (0.76; 0.87)	73	78	> 198
Mean (HU)	0.81 (0.75; 0.86)	62	86	> -483
Q.50 (HU)	0.79 (0.73; 0.84)	64	81	> −531
Kurtosis	0.78 (0.72; 0.83)	73	69	≤ 2.84
Skewness	0.77 (0.68; 0.80)	76	63	≤ 0.55
Consolidation ratio	0.75 (0.69; 0.80)	78	64	> 0.4
Max. diameter (mm)	0.72 (0.65; 0.78)	67	71	> 20.04
Volume (mm^3^)	0.71 (0.65; 0.77)	78	58	> 1,495
Volume (log)	0.71 (0.65; 0.77)	78	58	> 3.175
Average diameter (mm)	0.71 (0.64; 0.77)	76	61	> 15.9
Min. diameter (mm)	0.67 (0.60; 0.73)	61	68	> 13.3
Max./min. diam ratio	0.61 (0.54; 0.67)	37	83	> 1.74

The table summarizes the features in descending order of AUC magnitude. AUCs are presented with their (95% CI).

H1 or H2, N = 95 (42%).

H3, N = 132 (58%).

Q.50: CT attenuation value at the 50th percentile, Q.75: CT attenuation value at the 75th percentile, Q.875: CT attenuation value at the 87.5th percentile.

HU: Hounsfield unit.

Consolidation ratio: solid component maximum diameter/nodule maximum diameter.

Max.: maximum, Min.: minimum, Diam.: diameter.

**Table 3:** Main features and ROC-AUC performances for H1 vs H2 or H3 nodule classification (full dataset).

Parameter	AUC (95% CI)	Sensitivity	Specificity	Best Threshold
Q.875 (HU)	0.86 (0.81; 0.91)	61	100	> -277
IQR (HU)	0.84 (0.79; 0.89)	76	81	> 225
FPC1	0.82 (0.77; 0.87)	63	90	> −0.019
Q.75 (HU)	0.82 (0.77; 0.87)	59	94	> −393
SD (HU)	0.82 (0.77; 0.87)	76	77	> 172
Mean (HU)	0.82 (0.77; 0.87)	67	84	> −546
Q.50 (HU)	0.82 (0.77; 0.87)	83	71	> −656
Consolidation ratio	0.79 (0.73; 0.84)	70	84	> 0.31
Kurtosis	0.73 (0.67; 0.79)	38	100	≤ 2.35
Max. diameter (mm)	0.72 (0.65; 0.78)	57	81	> 20.3
Volume (mm^3^)	0.70 (0.63; 0.75)	51	84	> 2,601
Volume (log)	0.70 (0.63; 0.75)	51	84	> 3.41
Average diameter (mm)	0.71 (0.64; 0.77)	76	61	> 15.9
Min. diameter (mm)	0.70 (0.64; 0.76)	56	84	> 13
Skewness	0.65 (0.59; 0.71)	34	97	≤ 0.23

The table summarizes the features in descending order of AUC magnitude. AUCs are presented with their (95% CI).

H1, N = 21 (14%).

H2 or H3, N = 196 (86%).

Q.50: CT attenuation value at the 50th percentile, Q.75: CT attenuation value at the 75th percentile, Q.875: CT attenuation value at the 87.5th percentile.

HU: Hounsfield unit.

Consolidation ratio: solid component maximum diameter/nodule maximum diameter.

Max.: maximum, Min.: minimum.

#### Ordinal regression

At the previous step, volume (log), FPC1, and Q.875 were determined as the best candidates features for building a 3-class nodule classification model of 1) H1 vs H2 or H3 and 2) H1 vs H2 or H3, using an ordinal regression model with a cumulative logit and proportional odds.

First, diagnostic plots (ordinality plots, score residual plots for logistic models and smooth partial residual plots) showed that the ordinality and the proportional odds assumptions were acceptable for the features volume (log), FPC1 and Q.875 (Supplementary Information online, Supplementary Fig.S1 and S2 online). Additionally, tests on model deviances did not reveal significant differences in goodness-of-fit between our simple ordinal regression model with a cumulative logit and proportional odds and corresponding more complex models such as non-proportional odds cumulative logit models or partial proportional odds models.

**Figure 3:**
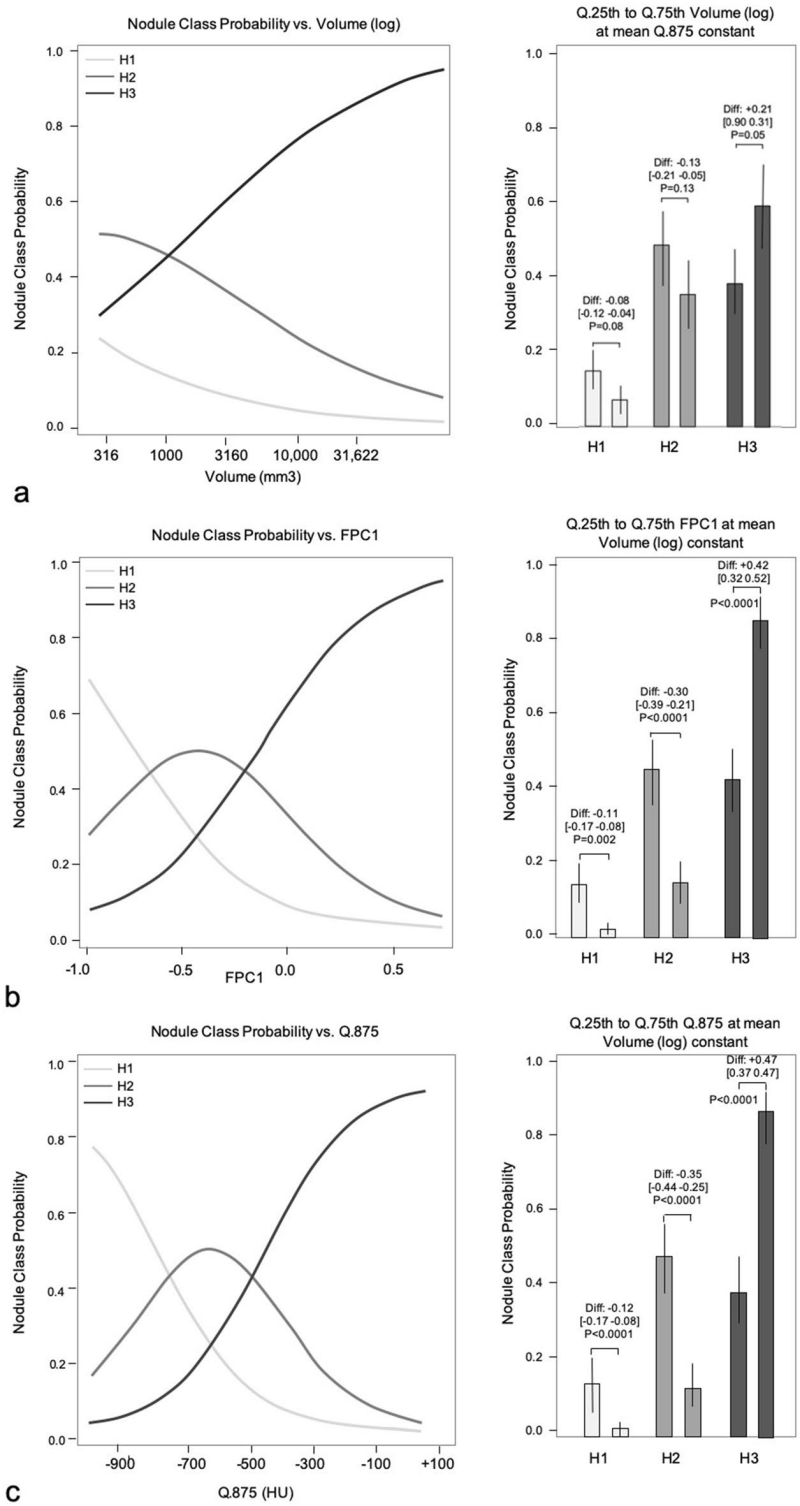
Class probability change with the model predictors. This figure represents the estimated probability for each nodule class (H1, H2, H3) varying with either the nodule volume (log) (a), the FPC1 value (b) and the Q.875 (c). Left: Change in class probabilities with the model predictors: a. Volume (log) (Model 1 & 2). b. FPC1 (Model 1) and c. Q.875 (Model 2). Right: Change in class probabilities between predictor 1st (25%) and 3rd (75%) quartiles for nodule volume, FPC1 and Q.875. Non-significant *P* values are omitted.

Then, two models based on 1) the nodule volume (log) (for Model 1 and Model 2) and 2) one CT attenuation feature (FPC1 for Model 1, and Q.875 for Model 2) were studied. Supplementary Fig. S3 online illustrates the dual contribution of the 2 features that were used in Model 1 (volume (log) and FPC1). Model 1 and Model 2 estimates (coefficient, odds ratios and intercepts) were evaluated on the full dataset and are presented in Table 4. For Model 1, after exponentiation of each feature coefficient, the odds ratios were 3.91 (2.02 to 7.78) for volume (log) and 38.23 (15.05 to 104.98) for FPC1. Consequently, for every 1-unit increase in volume (log), the odds of being H2 or H3 rather than H1 is increased 3.91 times. For every 1 unit increase in volume (log), the odds of being H3 rather than H2 or H3 is increased 3.91 times. Similarly, for every 0.1 unit increase in FPC1, the odds of being H2 or H3 rather than H1 is increased 3.82 times. For every 0.1 unit increase in FPC1, the odds of being H3 rather than H1 or H2 is increased 3.82 times. The same interpretation can be applied to Model 2 with the odds ratios: (for 1-unit increase) 3.49 for volume (log) and 1.0070 for Q.875 (a 100 units (HU) Q.875 increase is associated with a 70% odd increase). Additionally, the changes in class H1, H2 or H3 probabilities between the 25th percentile and 75th percentile of each predictor were computed and are illustrated in Fig. 3. The H1 class is significantly less likely to occur with an increased FPC1 (*P* = 0.002) or an increased Q.875 (*P* < 0.001) but not with an increased volume (*P* = 0.08). The H2 class is significantly less likely to occur with an increased FPC1 (*P* < 0.001) and an increased Q.875 (*P* < 0.001) but not with an increased volume (*P* = 0.13). Conversely, the H3 class is significantly more likely to occur with an increased FPC1 (*P* < 0.001) and Q.875 (*P* < 0.001) but not with an increased volume (*P* = 0.05).

**Table 4:** Model 1 (volume (log) + FPC1) and Model 2 (volume (log) + Q.875) estimates for the 3-class nodule classification using the ordinal regression model (full dataset).

	Coefficients, odds ratio and intercepts	*P* values (Wald test) for coefficients and intercepts
**Model 1: Class ~ volume (log) + FPC1**		
Volume (log)	C: 1.362 (0.690; 2.035) OR: 3.91 (2.02; 7.78)	< 0.001
FPC1	C: 3.644 (2.675; 4.619) OR: 38.23 (15.05; 104.98)	< 0.001
H1 vs H2 or H3 (Y ≤ H2)	I: −2.702	< 0.001
H1 or H2 vs H3 (Y ≤ H3)	I: −0.5208	0.003
**Model 2: Class ~ volume (log) + Q.875**		
Volume (log)	C: 1.251 (0.558; 1.943) OR: 3.493 (1.770; 7.098)	< 0.001
Q.875	C: 0.00695 (0.005; 0.009) OR: 1.0070 (1.005; 1.009)	< 0.001
H1 vs H2 or H3 (Y ≤ H2)	I: −2.8179	< 0.001
H1 or H2 vs H3 (Y ≤ H3)	I: −0.5197	0.003

The table presents the coefficients (C), the odds ratios (OR) and the intercepts (I) with their (95% CI) for Model 1 and Model 2.

#### Predictive (cross-validated) performances

Performance metrics of the ordinal regression models built at the previous step are presented in Table 5. Performance metrics were computed for the first (H1 vs H2 or H3) and for the second (H1 or H2 vs H3) cutoff points on the ordinal response scale. AUCs (from 0.83 to 0.87) and normalized Brier’s scores were slightly higher with Model 2 (28%) when compared to Model 1 (18%) for the first cutoff point and similar for the second cutoff point (36 and 37% respectively), with mean calibration error < 10% for both models.

**Table 5:** Predictive (cross-validated) performances for the 3-class nodule classification with Model 1 (volume (log) + FPC1) and Model 2 (volume (log) + Q.875) using the ordinal regression model.

Ordinal regression models	AUC1	AUC2	Normalized Brier’s score 1 (%)	Normalized Brier’s score 2 (%)	EAVG 1	EAVG 2
**Model 1. volume (log) + FPC1**						
Mean	0.83	0.85	18	36	0.060	0.075
2.5%	0.69	0.74	0	15	0.016	0.022
97.5%	0.97	0.96	45	57	0.091	0.125
**Model 2. volume (log) + Q.875**						
Mean	0.87	0.86	28	37	0.052	0.072
2.5%	0.75	0.76	2.5	15	0.017	0.016
97.5%	0.99	0.96	53	60	0.083	0.123

AUC1 and AUC2: AUC value for the first cutoff point (H1 vs H2 or H3) or the second cutoff point (H1-H2 vs H3).

Brier’s Score 1 and Brier’s Score 2: Brier’s score value for the first cutoff point (H1 vs H2 or H3) or the second cutoff point (H1 or H2 vs H3). Brier’s scores were normalized with range between 0 and 100%

EAVG1 and EAVG2: average probability calibration error value for the first cutoff point (H1 vs H2 or H3) or the second cut-off point (H1 or H2 vs H3).

### Comparison to other linear and non-linear classifiers

Predictive performances for 3-class nodule classification were also evaluated using alternative classifiers. A simple logistic regression model using either Model 1 predictors (volume (log) and FPC1) or Model 2 predictors (volume (log) and Q.875) and 2 cutoff points was the best classifier with similar classification performances compared to the ordinal regression models: AUCs ranging from 0.83 to 0.87, Brier’s scores from 18 to 27% (cutoff point 1) to 33% to 35% (cutoff point 2), and EAVG below 10% (Supplementary Table S2 online).

Besides logistic regression models, a 3-class linear discriminant analysis (LDA) was the most accurate classifier with AUC for class H1: 0.87, AUC for class H2: 0.72 and AUC for class H3: 0.85 using the nodule volume (log) and Q.875 (Model 2 predictors). The LDA with Model 1 predictors (volume (log) and FPC1) showed similar classification performances (AUC) except H1 classification where Model 2 was more accurate. A comparison of multiclass performances (AUC, sensitivity and specificity) between ordinal logistic regression models and LDA models is shown in Supplementary Table S3 online.

Despite the added computational burden, non-linear classifiers such as support vector machine with Gaussian or polynomial kernels, k-nearest neighbor or random forests, were not more accurate than logistic regression models when evaluated through cross-validation for the 3-class nodule classification. Because of space constraints their performances are not reported here.

## Discussion

In this study, we proposed two parsimonious models for the prediction of lung adenocarcinoma subtypes presenting as subsolid nodules on CT. Based on the ordinal regression method, these models provide a 3-class classification of invasiveness with discriminative power AUCs of 0.83 to 0.87 and predict the class probabilities with less than a 10% average error on independent testing sets. These 3-class performances are comparable to those from previous studies that only aimed at a 2-class classification of adenocarcinoma invasiveness^5,8,11,12^ and could be used to support medical decision making for the management of subsolid nodules. Detailed information on the effect of the predictors on the nodule class probabilities is derived from the ordinal regression models.

Logistic regression models have been successfully used to discriminate two classes of lung adenocarcinomas presenting as subsolid nodules according to their degrees of invasiveness^5,8,11,12^. However, the adenocarcinoma spectrum on histology covers more than two classes of nodules. Pre-invasive lesions (AAH and AIS) are morphologically distinct from minimally invasive lesions, and all these are distinct from invasive lesions in terms of prognosis and surgical management^3^. Ordinal regression models are a natural extension of binary logistic regression for 3-class problems. When underlying assumptions are verified, the proportional odds model adopted in this study offers very concise models with only one coefficient per predictor and two intercepts for a 3-class problem. Moreover, contrary to black box-type classifiers for very large datasets such as convolutional neural networks, binary logistic and ordinal regressions provide detailed insight on how the changes in the main CT features affect the different nodule class probabilities as shown in Fig. 3. These data could be valuable as an exploratory tool for identification of imaging biomarkers and for future development of decision support systems.

It is noticeable that the best performances for the 3-class nodule classification were obtained with only two predictors while more complex models were underperforming on testing set validation. These two predictors (volume and FPC1 for Model 1 or volume and Q.875 for Model 2) can be generated in a simple manner from the 3D nodule segmentation, now available on most clinical workstations, and from the histogram of CT attenuation values generated after the segmentation. Nodule volume (log) is the common predictor to Model 1 and Model 2. Nodule size, either assessed with the nodule maximum diameter or with the nodule volume, has been identified as a significant predictor of invasiveness in several studies that aimed at a binary classification of lung adenocarcinoma invasiveness^5,6,11,13^. In our study, it also only modestly correlated with the best CT attenuation predictors (R: 0.30). Its presence in the two final models was, therefore, expected. Nodule size is also used in various guidelines for the management of lung nodules incidentally discovered on CT, to individualize different management categories^14,15^. However, the univariate analysis revealed that most CT attenuation features had better class prediction than geometric features, including the nodule volume, and therefore should be included in the models. Additionally, Fig. 3 shows that a variation in nodule volume (log) between the 1st and the 3rd quartile is not associated with a significant change in class probability. Conversely, the same variation in nodule attenuation features (Q.875 or FPC1) is associated with a significant change in class probability for all classes. This finding illustrates the predominant weight of nodule attenuation features over geometric features for the prediction of invasiveness.

Among these CT attenuation features, the quantile-based feature Q.875 and the functional feature (extracted from the functional principal component analysis of the attenuation curves) FPC1 were finally retained to be included in Model 1 and Model 2. Q.875 values and other quantile variants have been used in previous predictive models^16,17^. The feature FPC1 is a data driven alternative to Q.875. Unlike Q.875, FPC1 does not pre-specify any arbitrary quantile threshold. Instead, it uses the whole set of CT attenuation curves of the dataset and attributes a numeric value to each curve relative to their shape inside the main mode of variation. Low FPC1 values can be interpreted as homogeneous low CT attenuation curves; they were found associated with H1 class. Large FPC1 values correspond to heterogeneous high attenuation curves, and were associated with H3 class (Supplementary Fig. S3 online and Fig. 3b). H2 class was located between both extreme classes in terms of FPC1 values. Model 2 that used the quantile-based Q.875 feature had slightly better classification performance for the separation of H1 nodules from nodules of higher invasiveness (H2 or H3) as compared to Model 1 based on FPC1. Q.875 itself was highly correlated with the FPC1 feature (R: 0.94). Knowing that the computation of Q.875 is very simple, it may be the first candidate for a translation to clinical practice. However, only FPC1 has the ability to provide an insight on the main mode of variation of CT attenuation profiles within the dataset and a data driven support for the interpretation of the predictor effect.

Binary logistic regression models with two cutoff points (1: H1 vs H2 or H3 and 2: H1 or H2 vs H3) showed similar performances (AUC: 0.82 to 0.87 and AUC: 0.84–0.85 respectively) compared to the ordinal regression models, however with an increased model complexity (2 coefficients per predictor instead of one for the ordinal regression). The alternative linear classifier LDA showed class discriminatory performances similar to those of the ordinal regression models for the classification of H1 nodules (AUC: 0.83 to 0.87) and H3 nodules (AUC: 0.85). LDA does not perform better than ordinal or binary logistic regression when predictors are not normally distributed, which is the case for both FPC1 and Q.875^18^. For H2 classification, the AUC was only 0.72. This lower value reflects the more challenging task of individualizing minimally invasive nodules with less extreme volume and CT attenuation values.

By visually assessing the size and the attenuation of a given persistent subsolid nodule, radiologists can empirically provide a first evaluation of its invasiveness, knowing that invasive nodules tend to be larger and with a higher CT attenuation as compared to non-invasive nodules^5,12,19,20^. However, this assessment is subjective in nature and prone to inter-observer variability. In a study performed on 54 nodules within the adenocarcinoma spectrum, Maldonado et al. found that the agreement between two radiologists for the differentiation between minimally invasive and invasive adenocarcinoma was only moderate (Cohen’s Kappa: 0.49). Moreover, only 36 cases (67%) of the cases were correctly categorized^21^. As a first step towards a more quantitative assessment, the size of solid component of the nodule has been proposed as a simple predictor of adenocarcinoma invasiveness^5,19,22^. Nevertheless, on one hand, the identification of a solid component in a given nodule is also prone to inter-observer variability^23,24^. On the other hand, the measurement of the size of a potential solid component with electronic calipers may vary between readers and display windows^25,26^, especially in complex part-solid lesions with more than one solid component. Quantitative models as the ones built in this study could complement the first subjective assessment performed by the radiologist and act as an objective support for medical decision-making, thereby opening the path for future more automated nodule classification tools available on clinical workstations, allowing for a quantitative assessment and histological prediction as part of a synoptic radiology report. Automated segmentation of the solid component of part-solid nodules may further improve these predictions^27^.

The histological categorization of lung adenocarcinoma subtypes according to their degree of invasiveness (preinvasive lesions: AAH and AIS, minimally invasive lesions, invasive lesions) was designed to ensure a common language between pathologists and a standardization of practice. Nevertheless, adenocarcinoma invasiveness is a continuous spectrum, with overlaps between the previously cited categories^28^. The gap between the accuracy of our models and a perfect, ideal model that would have an AUC of 1 may be due to technical limitations, but could also be a consequence of the histological categorization own imperfections.

Our study has several limitations. First, there was a selection bias in the cohort. Only subsolid nodules that were surgically resected and that were proven to belong to the adenocarcinoma spectrum were included. Therefore, our nodules are not a true representative reflection of the subsolid nodules that are discovered in routine practice. However, this study design provides a pathologically homogeneous sample of lung nodules. A further step would be to test our models in cohorts of subsolid nodules discovered incidentally or during lung cancer screening programs, during which they are commonly encountered^29^. Second, given that CT examinations were performed at two different centers over the course of more than a decade, different CT scanner units and CT protocols have been used. We only included patients with unenhanced CT examinations that were reconstructed with a 1-3 mm section thickness to limit technical variability. Additionally, our dataset mirrors the technical variability of CT examinations in routine practice. Therefore, the external applicability of our models may be enhanced thanks to this variability. Third, only 31 on the 227 lung nodules were type H1 nodules. Further studies with larger sample size would be needed to better evaluate the classification performances of H1 non-invasive nodules. Finally, besides size and CT attenuation, other features related to 3D texture or nodule shape were not evaluated in this study, knowing that the variability of acquisition, the voxel anisotropy and different slice thicknesses are a known challenge for texture-based features^30^. Automated texture or shape-based features and nodule volume growth on follow-up CT examinations should deserve special attention in future studies.

To conclude, simple ordinal regression models using a couple of predictors extracted from a subsolid nodule segmentation (volume (log) and a CT attenuation feature, either Q.875 or FPC1) can predict the nodule invasiveness class and its probability with acceptable performances in a 3-class context (AUC: 0.83 to 0.87 and average probability calibration error < 10%). This expands on previous results obtained for a 2-class nodule invasiveness classification where pre-invasive and minimally invasive classes were combined in a single group. Reliable multiclass prediction may eventually lead to an early CT based virtual pathology information to help the clinician with decision making. The predictive modelling approach adopted in this paper provides detailed insight on how the value of the main predictors contribute to the probability of invasiveness of cancer with the nodule and underlines the role of nodule CT attenuation in improving the ability to successfully classify the adenocarcinoma spectrum lesions. Testing the current models in fully independent datasets would validate their performances before translation to clinical practice.

## Methods

### Patients and nodules

This retrospective study followed the STROBE guidelines^31^ and was based on 2 cohorts from 2 academic centers: cohort 1 from the Beth Israel Deaconess Medical Center, Boston, MA, USA and cohort 2 from the Toronto General Hospital, Toronto, ON, Canada. The study protocol was approved by institutional review boards at each institution and informed consent was waived (center 1—DFCI protocol No. 17–110 and center 2—UHN protocol No. 15–9,691.3). For both cohorts, hospital databases were searched for all patients with a pathological diagnosis of primary lung adenocarcinoma post-surgical resection (inclusion periods: center 1—January 2005 to December 2018, center 2—January 2013 to August 2016 in center 2). Adenocarcinomas were included if: 1) an unenhanced pre-resection chest CT examination with a section thickness ≤ 3 mm was available, and 2) the adenocarcinoma presented as either a non-solid or a part-solid nodule on CT examinations. Adenocarcinomas were excluded if the adenocarcinoma could not be reliably identified on CT images. The 137 nodules from cohort 1 fulfilling the inclusion criteria were part of a radiologic-pathologic data repository of pulmonary adenocarcinomas used for previous publications^32−42^. Of the 90 nodules from cohort 2 fulfilling the inclusion criteria, 85 were part of a previous publication^11^. Thus, 227 nodules were included overall. Pathology work-up and adenocarcinoma classification into atypical adenomatous hyperplasia (AAH), adenocarcinoma in situ (AIS), minimally invasive adenocarcinoma (MIA) and invasive adenocarcinoma subtypes, has been described in a previous study^11^. CT acquisition and reconstruction parameters are described in Supplementary Information online. Patient, CT and nodule characteristics in both cohorts are shown in Supplementary Table S4 online.

### Solid component diameter measurement and nodule segmentation

All CT images were anonymized and transferred as DICOM images for analysis to a Vitrea workstation (Vital Images Inc, version 7.8, Minnetonka, MN, USA). First, the maximum diameter of the solid component was measured with electronic calipers in the lung window setting on the transverse CT section that displayed the largest solid component dimensions^14^, the diameter being reported as 0 for non-solid nodules. Then, each nodule was individually segmented. The segmentation was automatic after placing a seed in the center of the nodule but could be manually corrected if required. Vessels were excluded from the region of interest wherever possible. Solid component maximum diameter measurement and nodule segmentation were performed by one thoracic radiologist in each center respectively (C.d.M.-M. in center 1, A.O. in center 2).

### Extraction of imaging features

After nodule segmentation, both nodule geometric features and CT attenuation histograms were exported for further analysis. The following geometric features, automatically generated from the segmentation, were recorded: nodule minimum and maximum diameters, average diameter (average of minimum and maximum diameters), and nodule volume (expressed in logarithmic scale and named as volume (log)). The maximum to minimum ratio (maximum tumor diameter/minimum tumor diameter) and the consolidation ratio (solid component maximum diameter/nodule maximum diameter) were also computed. Additionally, CT attenuation features were generated from the nodule CT attenuation histograms. Standard parametric CT attenuation features included mean, standard deviation, skewness, and kurtosis. The following nonparametric CT attenuation features were also computed: Q.50 (median), Q.75 (at the 75% percentile), Q.875 (at the 87.5% percentile) and inter-quartile range (IQR). Finally, after converting the CT attenuation histograms into smooth curves, a functional principal component (FPC) analysis was applied to the entire dataset attenuation curves following Petersen & Muller’s method for attenuation distributions^43^. Both curve smoothing and FPC analysis methods have been described previously^11^.

### Statistical analysis

Statistical analysis was performed with R statistical program^44^. Specific R packages are described below.

#### Descriptive analysis

The normality of feature distributions was assessed using the d’Agostino-Pearson test. Normally distributed continuous variables are reported as means with their standard deviations. Non-normally distributed continuous variables are reported as medians with their lower and upper quartiles (IQR). Differences between categorical variables were assessed with a Chi-squared test. Differences between 2 groups of continuous variables were assessed with t-test or Mann–Whitney test, based on the normality test. For the comparison of > 2 groups of continuous variables, if the Levene test for the equality of variances was positive and the normality of the residuals was verified, one-way ANOVA with Sheffe post-hoc tests were applied. Otherwise, Kruskal–Wallis tests with Jonckheere trend tests were used, followed by Conover post-hoc tests. A two-sided *P* value < 0.05 was chosen to indicate a statistically significant difference.

#### Model building with ordinal regression

The aim was to build a 3-class nodule classification model: the class of pre-invasive nodules (AAH and AIS) was called H1, the class of MIA was called H2, and the class of invasive cancers was called H3.

The first step consisted of a feature selection. Selection of features was based on their individual ROC-AUC performance in the full dataset for nodule classification into both 1) H1 or H2 vs H3 and 2) H1 vs H2 or H3. Then, a confounder plot was computed using the R-library “Clumix”^45^, as previously described^46^. Built on a distinct approach that relies on variable similarity (see Supplementary Information Online), the confounder plot shows each feature based on its correlation with the response (H1, H2, H3 nodule class) and on its correlation with a reference feature. The reference feature can be chosen according to prior knowledge about its discriminant value and/or according to its individual AUCs in the study. Using this plot, features with the highest correlation with the response and the lowest correlation with the reference feature were selected for further models.

The second step consisted of building 3-class nodule classification models of 1) H1 or H2 vs H3 and 2) H1 vs H2 or H3 based on the previously selected features. The model building was performed using an ordinal regression model with a cumulative logit and proportional odds^47,48^. The use of this model was based on the premise that the 3 classes of nodules H1, H2 and H3 could be ordered according to associated risk and clinical significance. First, the ordinality and the assumptions underlying the ordinal regression model with a cumulative logit and proportional odds were verified using graphical methods and formal tests with custom code based on the R-library “rms”^49^ and “VGAM”^50^ (detailed in Supplementary Information online). Then, the goodness-of-fit of this simple ordinal regression model with a cumulative logit and proportional odds, and of corresponding more complex ordinal regression models (non-proportional odds cumulative logit models and partial proportional odds models) were compared with Chi-squared test on model deviances. Finally, the results of the ordinal model estimates were presented with their coefficients, odds ratios and intercepts. Each feature was previously centered. Additionally, to allow for a more intuitive interpretation, the changes in probabilities to be in class H1, H2 or H3 between the 25th percentile and the 75th percentile of the predictor of interest were also computed using a Monte Carlo simulation, keeping the other predictor of the model constant using Zelner’s method^51^. These changes are presented in nodule class probability plots for each final model predictor.

The third step was to assess the predictive performances of the previously built models. All predictive performance metrics reported in this study were evaluated using a 200-repeats Monte-Carlo cross-validation method with 80%-20% data splitting for each repeat using a stratified sampling to preserve the nodule class frequencies in binary or 3-class classification.

Performance metrics were computed for the first (H1 vs H2 or H3) and the second (H1 or H2 vs H3) cutoff points in the ordinal response scale. They included: AUCs, average probability calibration errors (EAVG) between predicted and observed probabilities and Brier’s scores. Brier’s score captures both nodule class discrimination and probability calibration aspects of the predictive performances^52^. It is computed as the mean square difference between the predicted probabilities and the value of the actual binary outcome. All Brier’s scores were rescaled by their maximum score for a non-informative model to let their range between 0% (worse) to 100% (best) following Steyerberg’s method^52^. All performance values (AUCs, EAVG and Brier’s scores) were computed using “val.prob” function of the “rms” R-library^49^.

#### Comparison to other linear and non-linear classifiers

Predictive performances for 3-class nodule classification were also evaluated with alternative classifiers using custom R-code and the R-library “Caret”^53^. Simple logistic regression, linear discriminant analysis (LDA), Naive Bayes, random forests, support vector machine, k-Nearest Neighbors were trained using accuracy as performance criteria on the same training dataset with tenfold cross-validation. Final models were evaluated and performances were averaged using a Monte-Carlo cross-validation method with 200 repeats.

## Supplementary information

Supplementary information

## Data Availability

The datasets generated and/or analyzed during the current study are available from the corresponding author on reasonable request.
