# Supplementary material for: Assessing invasiveness of subsolid lung adenocarcinomas with combined attenuation and geometric feature models
Source: Sci Rep. 2020 Sep 3;10:14585. doi: 10.1038/s41598-020-70316-3 (PMC7471897; doi:10.1038/s41598-020-70316-3)
Supplement: Supplementary file 1 — Supplementary information [file 41598_2020_70316_MOESM1_ESM.pdf]

Supplementary Information

Assessing invasiveness of subsolid lung adenocarcinomas with combined attenuation and geometric feature models

Constance de Margerie-Mellon* [1]

Ritu R. Gill [1]

Pascal Salazar [2]

Anastasia Oikonomou [3]

Elsie T. Nguyen [4]

Benedikt H. Heidinger [1,5]

Mayra A. Medina [6]

Paul A. VanderLaan [6]

Alexander A. Bankier [1]

1. Department of Radiology, Beth Israel Deaconess Medical Center and Harvard Medical School, Boston, MA, USA.
2. Vital Images, Minnetonka, USA.
3. Department of Medical Imaging, Sunnybrook Health Sciences Centre, University of Toronto, Canada.
4. Department of Medical Imaging, Toronto General Hospital, University of Toronto, Canada.
5. Department of Biomedical Imaging and Image-guided Therapy, Vienna General Hospital, Medical University of Vienna, Austria.
6. Department of Pathology, Beth Israel Deaconess Medical Center and Harvard Medical School, Boston, USA.

Correspondance to : Constance de Margerie-Mellon, constance.de-margerie@aphp.fr

nodules on CT examinations.

Supplementary Table S1. Results of the comparison of patient and CT protocol characteristics, and geometric and attenuation features for the 3 classes of nodules.

H1: atypical adenomatous hyperplasia and adenocarcinoma in situ
H2: minimally invasive adenocarcinoma
H3: invasive adenocarcinoma

Test for p-values: AOV: analysis of Variance, X2: Chi-2 test. KW: Kruskal-Wallis test, JT: Jonckheere test for trend. PH: Post-Hoc tests.

Max.: maximum, Min.: minimum, Diam.: diameter
Max.min.diam. ratio : maximum diameter/minimum diameter
Consolidation ratio: solid component maximum diameter/nodule maximum diameter
HU: Hounsfield unit
IQR: interquartile range
Q.50: CT attenuation value at the 50th percentile, Q.75: CT attenuation value at the 75th percentile, Q.875: CT attenuation value at the 87.5^{th} percentile

	**P-value**
**Age (years)**	0.49 (AOV)
**Sex**	
Male	P = 0.35 (X2)
Female	
**Smoking**	
No	P=0.29 (X2)
Yes	
**CT section thickness**	
1.0-1.5mm	P = 0.11(X2)
2.0-2.5mm	
3.0mm	
**Location**	
RUL	P = 0.18 (X2)
RML	
RLL	
LUL	
LLL	
**CT geometric features**	
Average Diameter (mm)	KW: < 0. 001. JT: <0. 001. PH-H1≠H3 & H2≠H3.
Max Diameter (mm)	KW: < 0. 001. JT: <0. 001. PH: H1≠H3 & H2≠H3.
Min. Diameter (mm)	KW: < 0. 001. JT: <0. 001. PH-H1≠H3 & H2≠H3.
Max.Min.Diam Ratio	KW: 0.02. JT: 0.006.PH: H2≠H3
Consolidation ratio	AOV: <0.001 (DF 2 224). PH: H1≠H2, H2≠H3, H1≠H3
**CT attenuation features**	
Mean (HU)	AOV: P < 0.001. PH: H1≠H2 H2≠H3 H1≠H3
Standard deviation (HU)	AOV: P < 0.001. PH: H1≠H2 H2≠H3 H1≠H3
Skewness	KW: P < 0. 001. JT: <0.001. PH-H1≠H3 & H2≠H3.
Kurtosis	KW: < 0. 001. JT: <0. 001. PH: H1≠H3 H2≠H3.
IQR (HU)	KW: P < 0. 001. JT: <0. 001. PH: H1≠H2, H2≠H3, H1≠H3
Q.50 (HU)	KW: < 0. 001. JT: <0. 001. PH: H1≠H, H2≠H3, H1≠H3
Q.75 (HU)	KW: < 0. 001. JT: <0. 001. PH: H1≠H2, H2≠H3, H1≠H3
Q.875 (HU)	KW: < 0. 001. JT: <0. 001. PH: H1≠H, H2≠H3, H1≠H3
Volume (log)	AOV: P < 0.001. PH: H2≠H3 H1≠H3
FPC1	KW: P < 0. 001. JT: <0. 001. PH: H1≠H2, H2≠H3 H1≠H3
FPC2	KW: P = 0.14.

Graphic review of the ordinality and proportional odds assumptions

The ordinality assumption for Model 1 and 2 was verified using an ordinality plot for ordinal regression first proposed by Harrel et al (Harrell, F. E. in *Regression Modeling Strategies: With Applications to Linear Models, Logistic and Ordinal Regression, and Survival Analysis, 2nd Edition Springer Series in Statistics*, 2015)

For each predictor, the means of the predictor stratified per class (H1, H2 and H3) is plotted together with the expected values of these means given that the proportional odds (PO) assumptions holds. Both the monotonic increase of means and the linearity of the lines connecting the 3 means support the ordinality hypothesis. The proximity between the observed stratified means (circles connected with solid lines) and the expected means under the PO assumption (dashed lines) is consistent with this assumption.

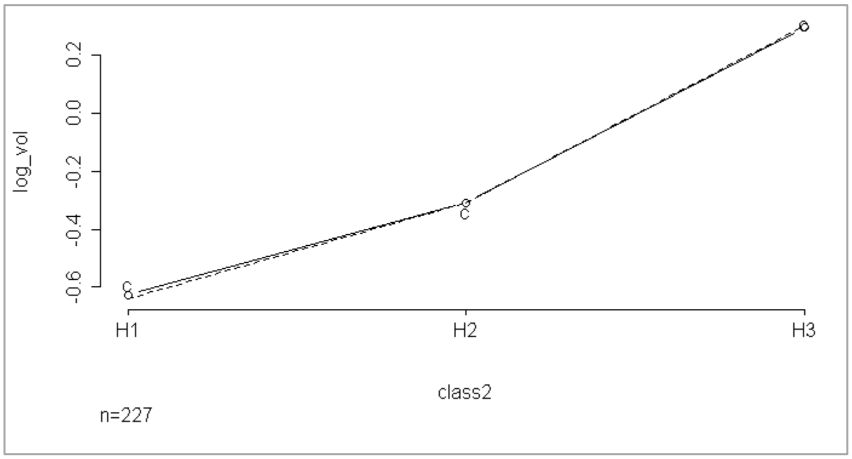

a.

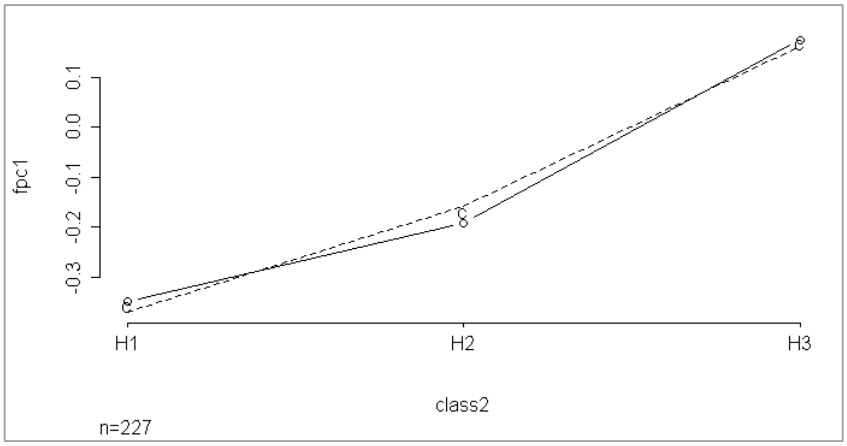

b.

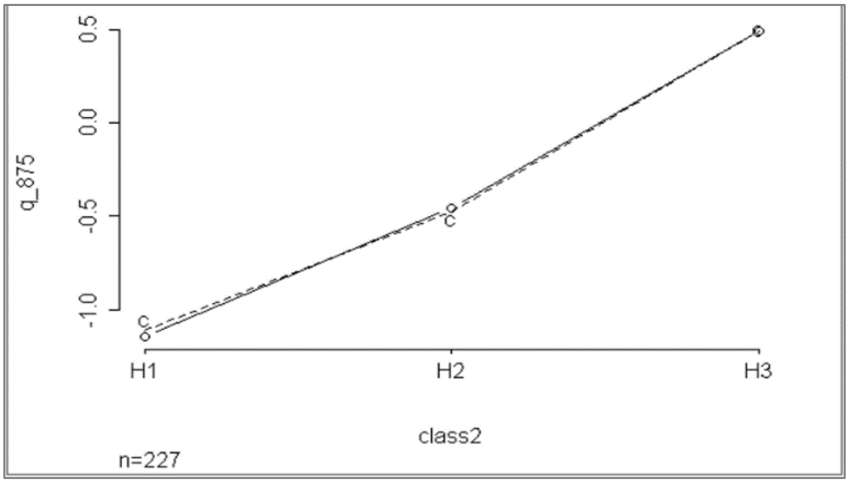

c.

Supplementary Figure S1. Ordinality plot for volume (log) (a), FPC1 (b) and Q.875 (c).

For the three predictors of Model 1 and 2 (volume (log), FPC1, Q.875) the stratified mean (circles) of each predictor and each nodule category H1, H2 & H3 follow a linear monotonic trend (solid lines) matching well the expected values of these means given that the proportional odds assumption holds (dashed lines).

Score residual plots for the PO model were computed by using the PO fitted model to predict the 2 binary events underlying our models: Y (nodule class) ≥ H2, and Y ≥ H3. See Harrell, 2015 for details.

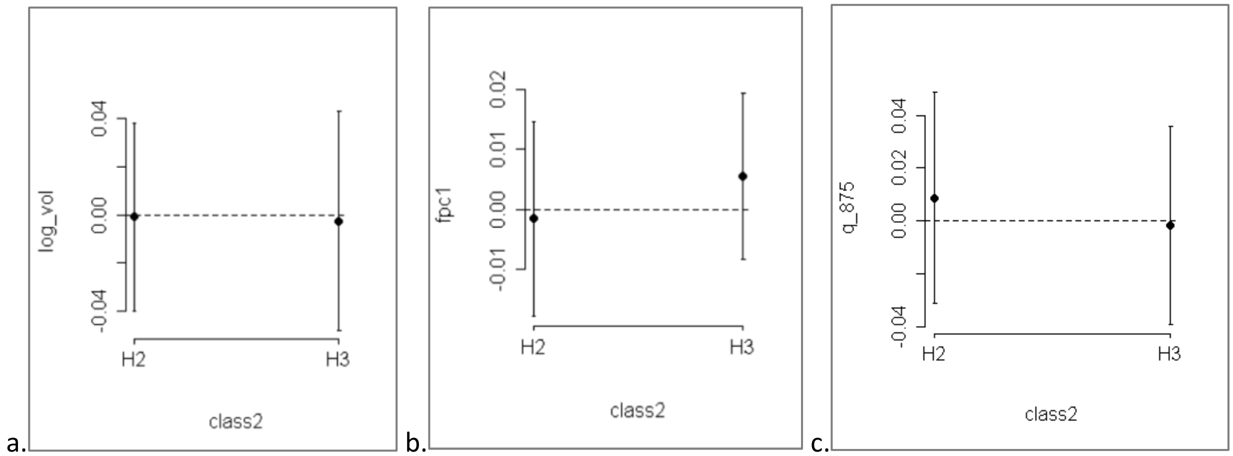

Supplementary Figure S2. Score residual plots for Volume (log) (a), FPC1 (b) and Q.875 (c).

For each cutoff points of the ordinal response (H2: response Y is H2 or H3, H3: response Y is H3). The PO assumption holds very well for volume (log) and is acceptable for both FPC1 and Q.875 predictors (the 2 black dots representing the mean residuals at each cutoff point are well aligned with the dash line assuming that the PO holds.

Besides the above graphical verification of PO assumption, the ordinal logistic models M1 (nodule class ~ volume (log) + FPC1) and model 2 (nodule 3-class ~ volume (log) + Q.875) with PO were tested against the same cumulative logistic models without PO assumptions or with partial PO assumptions (PO on volume, or FPC1 or Q.875 only) using the vglm function of the “VGAM” R-library. Chi-square tests using the model deviances did not find significant differences in goodness-of-fit between PO models and more complex non-PO or partial-PO models. However, Model 2 was found to have a significantly goodness-of-fit expressed as a lower deviance (312.9) compared to the Model 1 (326.4): chi-squared test P<0.0001) indicating a higher performance of Q.875 compared to FPC1 predictor.

Supplementary Figure S3. Distribution of nodules according to the nodule volume and the CT attenuation feature FPC1.

The 2D density map of the whole nodule dataset (227 cases) shows each nodule case according to their FPC1 and volume values. The three nodule classes H1, H2 and H3 occupy different positions in the plot with low FPC1 and small volume associated with the non-invasive H1 class, while there is an increase of minimally invasive H2 cases and invasive H3 cases densities for high FPC1 and large nodule volumes.

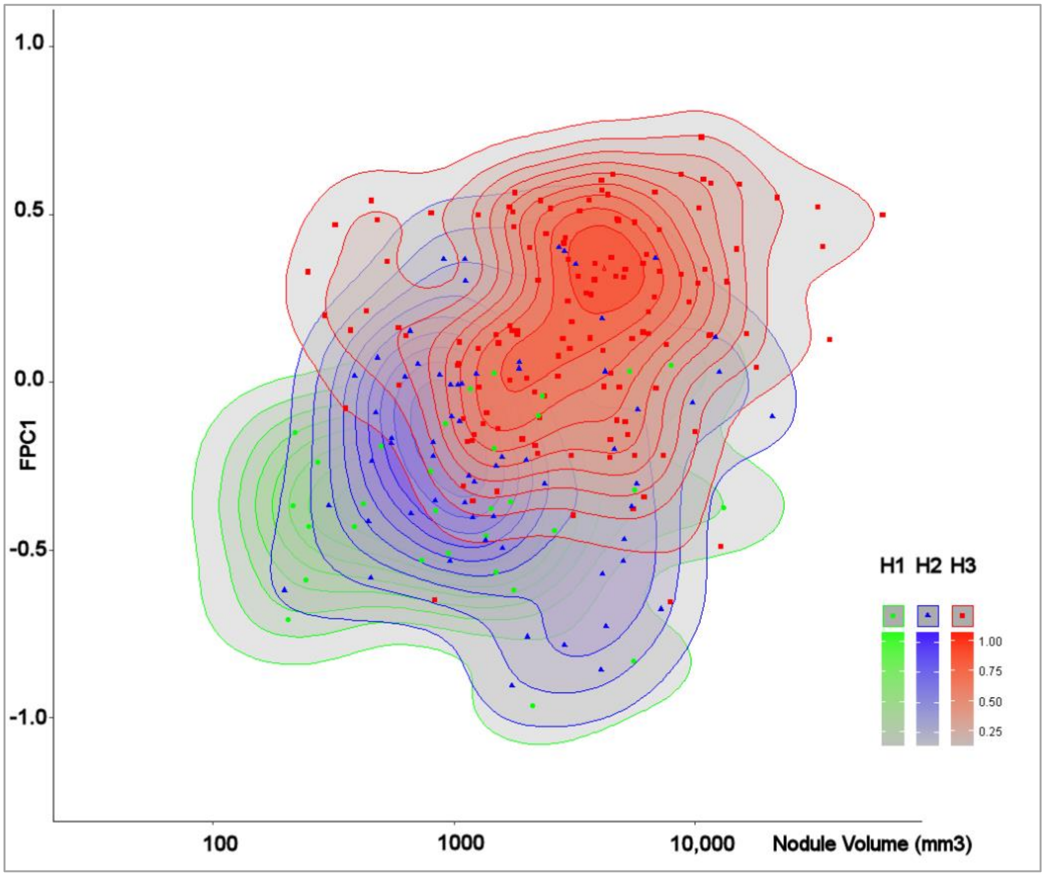

Supplementary Table S2. Predictive (cross-validated) performances for the 3-class nodule classification using the linear regression model.

AUC1 and AUC2: AUC values for the first cutoff point (H1 vs H2 or H3) or the second cutoff point (H1-H2 vs H3).
Brier's Score 1 and Brier's Score 2: Brier's score values for the first cutoff point (H1 vs H2 or H3) or the second cutoff point (H1 or H2 vs H3). Brier's scores were normalized with range between 0% and 100%.
EAVG1 and EAVG2: average probability calibration error value for the first cutoff point (H1 vs H2 or H3) or the second cut-off point (H1 or H2 vs H3).

Logistic regression models	AUC1	AUC2	Normalized Brier's Score 1	Normalized Brier's Score 2	EAVG 1	EAVG 2
**Model 1. Volume (log)+FPC1**						
**Mean**	0.83	0.84	18%	33%	0.060	0.074
**2.5%**	0.66	0.74	0%	12%	0.015	0.014
**97.5%**	0.98	0.94	47%	55%	0.101	0.129
**Model 2. Volume (log)+Q.875**						
**Mean**	0.87	0.85	27%	35%	0.057	0.072
**2.5%**	0.76	0.75	0%	13%	0.006	0.011
**97.5%**	1.00	0.95	58%	57%	0.096	0.013

Supplementary Table S3. Predictive (cross-validated) performances for 3-class nodule classification with Model 1 and Model 2 using ordinal logistic regression (ORD) and linear discriminant analysis (LDA).

Sensitivities (Sens.) and specificities (Spec.) are calculated comparing each factor level to the remaining levels (i.e. "one versus all" approach). All performances are presented with their (95% CI).

Ordinal Logistic Regression models	H1 Nodules	H2 Nodules	H3 Nodules
**Model ORD 1. Volume (log) + FPC1**			
**Ordinal Logistic Regression Volume (log) + FPC1**	AUC: 0.83 (0.66;0.95)<br>Sens.: 45% (04;100)<br>Spec.: 89% (85;94)	AUC: 0.72 (0.54;0.87)<br>Sens.: 48% (25;75)<br>Spec.: 80% (72;89)	AUC: 0.85 (0.71;0.95)<br>Sens.: 78% (68;87)<br>Spec.: 79% (58;100)
**Model ORD 2. Volume (log) + Q.875**			
**Ordinal Logistic Regression Volume (log) + Q.875**	AUC: 0.87 (0.73;0.96)<br>Sens.: 58% (08;100)<br>Spec.: 90% (86;95)	AUC: 0.72 (0.56;0.85)<br>Sens.: 47% (22;73)<br>Spec.: 80% (71;88)	AUC: 0.85 (0.73;0.95)<br>Sens.: 80% (70;89)<br>Spec.: 78% (59;100)
**Model LDA 1. Volume (log) + FPC1**			
**LDA Volume (log) + FPC1**	AUC: 0.83 (0.69;0.97)<br>Sens.: 43% (05;100)<br>Spec.: 87% (85;90)	AUC: 0.72 (0.58;0.86)<br>Sens.: 45% (25;67)<br>Spec.: 80% (72;89)	AUC: 0.85 (0.75;0.96)<br>Sens.: 77% (69;87)<br>Spec.: 77% (58;100)
**Model LDA 2. Volume (log) + Q.875**			
**LDA Volume (log) + Q.875**	AUC: 0.87 (0.76;0.99)<br>Sens.: 58% (05;100)<br>Spec.: 90% (86;95)	AUC: 0.72 (0.58;0.87)<br>Sens.: 46% (23;67)<br>Spec.: 80% (71;88)	AUC: 0.85 (0.75;0.96)<br>Sens.: 80% (69;89)<br>Spec.: 78% (60;100)

CT scanner parameters

Given the bi-centric design of the study, various CT scanner units were used (for Cohort 1: LightSpeed VCT and LightSpeed Pro, GE Healthcare, Waukesha WI, USA; Discovery 750HD, GE Healthcare, Waukesha WI, USA; Aquilion One, Canon Medical Systems, Otawara, Japan; Somatom Definition, Siemens Healthcare, Erlangen, Germany; for Cohort 2: Aquilion One, Canon Medical Systems, Otawara, Japan). All CT examinations were performed over the entire thorax, at full suspended inspiration, and with the patient in supine body position, and no contrast medium injection was used. The CT studies were completed using dose modulation with 120–135 kVp. All images were reconstructed with a standard kernel and visualized in lung window settings (mean −500 HU, width 1500 HU). Reconstruction section-thickness varied from 1 to 3mm.

Supplementary Table S4. Comparison of Cohort 1 and Cohort 2.

Normally distributed continuous features are shown as mean±standard deviation, non-normally distributed features as median (interquartile range). Categorical features are shown as number (%).

Consolidation ratio: solid component maximum diameter/nodule maximum diameter

	Entire cohort	Cohort 1 (BIDMC)	Cohort 2 (TGH)	Differences between the 2 cohorts
**Number of tumors**	227	137	90	NA
**Number of patients**	206	132	74	NA
**Clinical parameters**				
- Age (years)	66±10	68±9	64±10	*P*=.003
- Gender<br>o Female<br>o Male	 148 (72%)<br>56 (28%)	 98 (74%)<br>34 (26%)	 50 (69%)<br>22 (31%)	*P*=0.464
- Smoking history<br>o Former or current smoker<br>o Never smoker	 145 (71%)<br>59 (29%)	 94 (71%)<br>38 (29%)	 51 (71%)<br>21 (29%)	*P*=0.955
**Histology**				
- Adenomatous atypical hyperplasia/ adenocarcinoma in situ<br>- Minimally invasive<br>- Invasive adenocarcinoma	31 (14%)<br><br><br>64 (28%)<br>132 (58%)	12 (9%)<br><br><br>43 (31%)<br>82 (60%)	19 (21%)<br><br><br>21 (23%)<br>50 (56%)	*P*=.172
**CT parameters**				
- CT section thickness<br>o ≤1.25mm<br>o 2-2.5mm<br>o 3mm	 121 (53%)<br>17 (8%)<br>89 (39%)	 116 (85%)<br>16 (12%)<br>5 (4%)	 5 (6%)<br>1 (1%)<br>84 (93%)	*P*<.0001
- CT to surgery interval (days)	56 (34;81)	48(28;71)	72 (53;94)	*P*<.0001
**Tumor characteristics on CT**				
- Tumor morphology<br>o Non-solid<br>o Part-solid	 62 (27%)<br>165 (73%)	 47 (34%)<br>90 (66%)	 15 (17%)<br>75 (83%)	*P*=.004
- Tumor location<br>o RUL<br>o RML<br>o RLL<br>o LUL<br>o LLL	 77 (34%)<br>12 (5%)<br>37 (16%)<br>69 (30%)<br>32 (14%)	 43 (31%)<br>7 (5%)<br>22 (16%)<br>42 (31%)<br>23 (17%)	 34 (38%)<br>5 (6%)<br>15 (17%)<br>27 (30%)<br>9 (10%)	*P*=.650
- Maximal diameter (mm)	21 (15;28)	22 (17;29)	19 (14;27)	*P*=.130
- Consolidation ratio	0.43 (0;0.63)	0.38 (0;0.52)	0.52 (0.28;0.80)	*P*<.0001
- Volume (mm^3)	2239 (1098;5155)	2380 (1159;5411)	1939 (873;4762)	*P*=.222

Similarity measurements performed for the confounder plot (Fig. 2)

The following similarity measurements for each category of features were chosen for the confounder plot:

- Quantitative vs. quantitative or ordinal feature: absolute Spearman correlation coefficient (for instance Q.875 versus Nodule Class)
- Quantitative or ordinal vs. binary feature: absolute Goodman and Kruskal's gamma coefficient (for instance Smoking versus Nodule Volume). The Goodman & Kruskal gamma rank coefficient for quantitative/binary or binary/binary variable pairs is defined as $S_{ij} = |(n_c - n_d)/(n_c + n_d)|$ with n_c and n_d number of concordant and discordant pairs of observations i and j.

Verification of the assumptions underlying the proportional odds model

The ordinal logistic regression with proportional odds model can be defined using the standard formulation (Agresti, A. *Analysis of Ordinal Categorical Data. Second edition.* Wiley, Hoboken, NJ, USA, 2010).

for an ordinal response y with c categories and x the explanatory feature. We model P(y≤j), j=1,2,.,c-1, using logits.

$$logit[(Py \leq j)] = \log\frac{P(y \leq j)}{P(y > j)} = \alpha_j + \beta x, j = 1, .., c-1$$

With j: cutoff point index (in our case j=1 for H1 vs. H2 H3, and j=2 for H1 H2 vs. H3), α: Intercept associated with the cutoff point j and β: Coefficient associated with the predictor x. The logit is the logarithm of the odds (p/1-p). with p: probability of the negative outcome ("H2 H3" or "H3" depending on the cutoff point).
